# The impact of GLP-1 receptor agonist liraglutide on blood pressure profile, hydration, natriuresis in diabetic patients with severely impaired kidney function

**DOI:** 10.1038/s41598-024-55724-z

**Published:** 2024-02-29

**Authors:** Małgorzata Wajdlich, Michał Nowicki

**Affiliations:** Department of Nephrology, Hypertension and Kidney Transplantation, Central University Hospital, Medical University of Lodz, Pomorska 251, 92-213 Lodz, Poland

**Keywords:** Liraglutide, Diabetic kidney disease, Blood pressure, Natriuresis, Renin–angiotensin–aldosterone system, Chronic kidney disease, Hypertension

## Abstract

Chronic treatment with GLP-1R agonists may moderately lower blood pressure due to increased natriuresis and RAAS inhibition. Short-term effect of these drugs on blood pressure may be opposite and its mechanism remains unclear. We investigated the effect of a single dose of liraglutide on diurnal blood pressure profile, natriuresis, hydration and serum concentration of renin, aldosterone and atrial natriuretic peptide (ANP) in diabetic kidney disease (DKD). 17 patients with eGFR < 30 ml/min/1.73 m^2^ and 17 with > 60 ml/min/1.73 m^2^ received in a random order a single subcutaneous dose 1.2 mg liraglutide and placebo with subsequent 24 h blood pressure and natriuresis monitoring. Before and after each medication thoracic fluid index and plasma renin, aldosterone and ANP were also assessed. The blood pressure load in the daytime and nighttime were significantly increased after liraglutide compared to placebo in patients with eGFR < 30 ml/min/1.73 m^2^. In patients with eGFR > 60 ml/min/1.73 m^2^ the changes of arterial pressure were comparable, while the morning surge was significantly reduced after liraglutide compared to placebo. After liraglutide 24 h urine sodium excretion increased in both groups vs. placebo (*p* < 0.001), the effect was greatest in subjects with eGFR > 60 ml/min/1.73 m^2^. Plasma ANP increased after liraglutide in both groups, most in patients with eGFR < 30 ml/min/1.73 m^2^ group. Plasma aldosterone (*p* = 0.013) and thoracic fluid index (*p* = 0.01) decreased after liraglutide compared to placebo (*p* = 0.013 and *p* + 0.01, respectively. Plasma renin concentration remained unchanged. In severe chronic kidney disease liraglutide induces a transient increase of blood pressure due to reduced natriuresis. The natriuretic effect of liraglutide in DKD may be related to increased ANP and decreased aldosterone secretion.

## Introduction

Cardiovascular complications are the major cause of mortality in patients with diabetic kidney disease (DKD)^1^. Arterial hypertension has been recognized as a main risk factor for cardiovascular events and is highly prevalent in patients with DKD^2^. An impaired sodium excretion leading to fluid retention and increased activity of the renin-angiotensin- aldosterone system (RAAS) are the major pathogenic factors in the development of hypertension in DKD^3^.

High blood pressure is closely related to increased intravascular fluid volume in DKD^4^. Hyperglycemia and subsequent glycosuria increase the activity of sodium glucose cotransport-2 (SGLT-2), resulting in increased sodium reabsorption in the proximal tubule and fluid retention^5^. Hyperinsulinemia and insulin resistance may also contribute to the increased sodium reabsorption^5^. On the other hand the progression of chronic kidney disease (CKD) with a loss of nephrons shifts the pressure-natriuresis curve to the right^6^.

A disproportionate activation of the renin–angiotensin–aldosterone system has been also found to contribute to the development of hypertension in both non-diabetic and diabetic CKD. The hyperreninemia in chronic kidney disease develops mainly in response to ischemia of the kidneys caused by fibrosis and scarring^6^. There are several mechanisms leading to an imbalance of RAAS activity in diabetes. A high glucose concentration directly stimulates the local production of angiotensin II and enhances the tissue response to intrarenal RAAS activation^7^.

The complex pathogenesis of arterial hypertension in DKD may often hinder an optimal blood pressure control in this group of patients. Some new antidiabetic drug classes may decrease blood pressure and stimulate natriuresis^8^. It has been suggested that GLP-1 receptor agonists (GLP-1RA) may possess a blood pressure lowering effect. The meta-analysis of six trials of liraglutide showed a mean systolic blood pressure (SBP) reduction of 2.5 mmHg within 2 weeks of treatment^9^. Interestingly, while nearly all studies concluded that GLP-1RAs significantly reduced SBP, diastolic blood pressure (DBP) was not significantly affected^10^. A reduction of blood pressure during GLP-1 treatment was seen prior to any changes in body mass which may suggest that the effect can be directly related to GLP-1R agonist action. It was also postulated that the blood pressure lowering effect of GLP-1R agonists may be mediated by a direct vasodilatory effect and/or increased natriuresis and the inhibition of the renin–angiotensin–aldosterone system (RAAS)^8^. On the other hand the effects of GLP-1R agonists on BP vary both acutely and with chronic administration. Short-term effects of these drugs on blood pressure may be opposite but their mechanism is unclear. A number of studies involving acute infusion or administration (hours), mostly in healthy adults, have demonstrated either no change or an increase in BP, accompanied with increase of heart rate and cardiac output as a one of possible reasons of hypertensive effect of GLP-1RA^8^. We have also reported the hypertensive effect of liraglutide in patients in advanced stages of DKD in our previous publication^9^. Liraglutide administration in these patients induced increase of blood pressure due to increase of cardiac output secondary to acceleration of heart rate associated with sympathetic predominance. The vasodilatory effect of liraglutide was preserved only in earlier CKD.

In this paper we focused on the effect of a single dose of liraglutide and placebo on 24 h blood pressure profile, hydration, natriuresis and plasma concentration of renin, aldosterone and atrial natriuretic peptide in patients with diabetic kidney disease and impaired kidney excretory function.

### Patients and methods

The prospective single-dose cross-over double-blind placebo-controlled study included 34 patients divided into subgroups with two different degrees of renal function impairment, i.e. 17 patients with eGFR below 30 ml/min/1.73 m^2^ and 17 patients with eGFR above 60 ml/min/1.73 m^2^. Glomerular filtration rate was calculated using CKD-EPI equation. The trial was conducted in compliance with the Good Clinical Practice guidelines and the ethical principles stated in the Declaration of Helsinki. All patients that were included in this research work gave their written informed consent and the study protocol was approved by the Ethics Committee on Human Research of Medical University of Lodz, reference number of approval RNN/25/13/RE.

The inclusion criteria included: type 2 diabetes mellitus, eGFR below 30 ml/min/1.73 m^2^ or above 60 ml/min/1.73 m^2^, stable kidney function (± 5%) within three months before the study, the age > 18 years and < 80 years. The exclusion criteria comprised: diabetes type 1, acute kidney injury, history of solid organ or bone marrow transplantation, proteinuria over 2 g per 24 h, glycated hemoglobin (HbA1C) > 10%, history of pancreatitis, liver failure, allergic reaction to GLP-1R agonists, inflammatory bowel diseases, gastroparesis, nausea, vomiting, diarrhea within one month before the study, thyroid disease, pregnancy, heart failure NYHA III-IV, atrial fibrillation, atrial flutter, atrioventricular block, unstable angina, systolic blood pressure > 180 mmHg, diastolic blood pressure > 120 mmHg, confusion, psychiatric disorders, incompliance. All chronic antihyperglycemic and hypotensive medication as well as diuretics had not been changed for at least one month before the study and allowed to be continued in the same dose during the study. To avoid a possible effect of food and water intake and physical activity, the meal plan and total daily fluid intake as well as physical activeness were kept constant during the study and the patients received a dietary advice with provided by a renal dietitian before the study commenced. All the meals provided to the patients were prepared under a supervision of a dietician and contained a controlled sodium amount of 3 g/day. Baseline characteristics of the study group are shown in Table 1.

**Table 1:** Baseline characteristics of the patients with eGFR > 60 and < 30 ml/min/1.73 m^2^.

	eGFR < 30 ml/min/1.73 m^2^	eGFR > 60 ml/min/1.73 m^2^	*p*-value
Mean eGFR (ml/min/1.73 m^2^)^a^	25.1 ± 3.4	65.6 ± 4.6	< 0.0001
Age (years)^a^	68 ± 6	64 ± 9	0.2
Sex (F-female, M-male)	F 29.4% (n = 5) M 70.6% (n = 12)	F 64.7% (n = 11) M 35.3% (n = 6)	0.04
BMI (kg/m^2^)^a^	32.1 ± 3.9	31.3 ± 6	0.05
Duration of diabetes (years)^a^	16 ± 5	10 ± 4	0.001
Insulin therapy (%)	88.2% (n = 15)	29.4% (n = 5)	0.0005
HbA1c (%)^b^	7.3 (5.9–9.7)	6.8 (5.8–8.2)	0.005
Ischemic heart disease (%)	94.1% (n = 16)	64.7% (n = 11)	0.03
History of cardiovascular events (%)	47% (n = 8)	5.9% (n = 1)	0.02
History of smoking (%)	58.8% (n = 10)	17.7% (n = 3)	0.01
Duration of hypertension (years)^b^	16 (10–20)	10 (8–15)	0.01
The number of antihypertensive drugs^c^	4 (3–6)	4 (3–5)	0.01
ACE inhibitor	21.4% (5/17)	41.1% (7/17)	0.47
Angiotensin II receptor blocker	35.3% (6/17)	52.9% (9/17)	0.3
Spironolactone	0% (0/17)	5.9% (1/17)	0.3
Diuretics	100% (17/17)	88.2% (15/17)	0.14
Centrally-acting antihypertensives	17.7% (3/17)	23.5% (4/17)	0.67
Baseline SBP (mmHg)^a^	143 ± 14	132 ± 14	0.04
Baseline DBP (mmHg)^a^	75 ± 7	74 ± 8	0.74

BMI, body mass index; HbA1c, glycated hemoglobin; ACE, angiotensin converting enzyme; SBP, systolic blood pressure; DBP, diastolic blood pressure.

^a^Mean ± standard deviation.

^b^Median with interquartile range (IQR).

^c^Median with (min.-max) range.

Each subject received in a random order a single subcutaneous dose of 1.2 mg liraglutide and placebo at an interval of 48 h, with subsequent clinical follow-up in the metabolic ward, during which blood pressure was monitored for the following 24-h and 24 h urinary samples were collected to assess sodium excretion. Blood pressure and heart rate were monitored noninvasively using BR-102 plus ABPM (ambulatory blood pressure monitoring) system (Schiller, Baar, Switzerland). The device recorded blood pressure and heart rate in 15-min intervals during the day and 30-min intervals during the night. The diurnal periods, e.g. daytime activity and nighttime rest were determined based on activity diaries filled by the patients. The following parameters were derived from 24-h ABPM readings for blood pressure: mean systolic blood pressure (SBP) and diastolic blood pressure (DBP) over the 24-h period, SBP and DBP during day-time (awake) and night-time periods (asleep), mean arterial pressure (MAP) and mean pulse pressure (PP) over 24-h, day-time and night-time periods, BP load (percentage of ABPM readings > 135/85 mmHg during the waking period and 120/70 mmHg during sleeping period) and average deviation index (ADI), obtained by adding the percentage 24-h systolic deviation and 24-h diastolic deviation divided by two. Besides based on ABPM readings the night blood pressure fall (NBPF) and morning surge (MS) were determined. The night blood pressure fall was calculated according to the formula: NBPF = (Day-time MAP–Night-time MAP)/Day-time MAP × 100%. The morning surge was defined as the difference between the morning pressure during the first 2 h after awakening and the average of the lowest nighttime BP.

Additionally, before and after each medication the thoracic fluid index was measured with bioimpedance cardiography (Physioflow Enduro, Manatec Biomedical, France) and blood samples were collected for the measurement of plasma bioactive renin, aldosterone and atrial natriuretic peptide (ANP). Serum ANP was measured with immunoenzymatic commercial assay (Cloud Clone Corp, Huston, USA) and plasma aldosterone and renin concentrations with immunoenzymatic method (Diametra Corp, Spello, Italy). The samples were taken before and 11 h after study drug administration—at the time of peak blood concentration of the liraglutide^11,12^.

The statistical analysis was carried out with Statistica 13.1 software (StatSoft Poland, Cracow, Poland). Shapiro–Wilk test was used to check the normality of the variable distribution. A t-test for independent samples was used for comparisons of means between two groups for normally distributed variables. Student’s paired t-test was used to determine within group differences between pre- and post-intervention. Chi^2^ test was used to analyze categorical variables. If the data did not follow the normal distribution, the non-parametric tests were used, i.e. the Mann–Whitney U test for independent samples and the Wilcoxon signed rank test for paired samples. Pearson or Spearman correlation coefficient was calculated to assess the association between the variables depending on normality of their distribution. *P* < 0.05 was taken as statistically significant. The data are presented as mean ± SD or median and 25%-75% interquartile range (IQR). 95% Confidence intervals (95% Cl) were calculated for the changes of the variables after the administration of the study drug or placebo.

## Results

The mean daytime as well as nighttime diastolic blood pressure and mean arterial pressure were significantly higher after liraglutide injection compared to placebo but the differences were significant only in patients with eGFR < 30 ml/min/1.73 m^2^ (Table 2).

**Table 2:** Systolic, diastolic, mean arterial pressure, pulse pressure, systolic and diastolic blood pressure load recorded for 24 h after the administration of liraglutide and placebo in patients with eGFR < 30 ml/min and > 60 ml/min.

Study group	eGFR < 30 ml/min/1.73 m^2^		*p* value	eGFR > 60 ml/min/1.73m^2^		*p*-value
Parameter	Placebo	Liraglutide		Placebo	Liraglutide	
24 h SBP^1^ (mmHg)	140.7 ± 11.9	145.2 ± 13.8	***p*** **= 0.03**	133.9 ± 13.4	132 ± 14.5	*p* = 0.41
24 h DBP^1^ (mmHg)	74.8 ± 7.6	78.9 ± 7.1	***p*** **= 0.001**	74.8 ± 9.3	74.2 ± 7.3	*p* = 0.57
24 h MAP^1^ (mmHg)	97.8 ± 8.1	102.4 ± 8.6	***p*** **= 0.003**	97.8 ± 10.8	95.7 ± 8	*p* = 0.35
24 h PP^1^ (mmHg)	65.9 ± 10.2	66.4 ± 10.9	*p* = 0.7	59.2 ± 10.9	57.8 ± 13.6	*p* = 0.46
24 h SBP load^1^ (%)	59.8 ± 21.1	68.5 ± 19.2	***p*** **= 0.04**	51.5 ± 27.5	43.5 ± 2.7	*p* = 0.09
24 h DBP load^2^ (%)	15 (9–25)	22 (20–40)	***p*** **= 0.002**	15 (8–26)	11 (7,24)	*p* = 0.19
Daytime SBP^1^ (mmHg)	141.6 ± 12.3	146.4 ± 13.7	*p* = 0.05	136 ± 13.7	134 ± 14.9	*p* = 0.49
Daytime DBP^1^ (mmHg)	75.8 ± 8.5	79.9 ± 7.2	***p*** **= 0.018**	77 ± 9.1	76.3 ± 8.8	*p* = 0.83
Daytime MAP^1^ (mmHg)	98.8 ± 8.7	103.4 ± 8.1	***p*** **= 0.016**	97.8 ± 10.8	95.7 ± 8	*p* = 0.35
Daytime PP^1^ (mmHg)	66.1 ± 10.4	66.5 ± 11.1	*p* = 0.76	59.1 ± 10.8	57.2 ± 13.6	*p* = 0.39
Daytime BP load SBP^1^ (%)	5.6 ± 23.2	62.6 ± 23.1	*p* = 0.06	43.5 ± 27.7	35.7 ± 29.1	*p* = 0.18
Daytime BP load DBP^2^ (%)	6 (4–24)	18 (15–39)	***p*** **= 0.01**	17 (8–24)	7 (6–26)	*p* = 0.69
Nightime SBP^1^ (mmHg)	1364 ± 13.5	142.1 ± 18	*p* = 0.13	129 ± 16.8	127 ± 16.3	*p* = 0.48
Nightime DBP^1^ (mmHg)	71.4 ± 8.3	75.8 ± 10	***p*** **= 0.02**	68.8 ± 9.9	70.19 ± 7.3	*p* = 0.57
Nightime MAP^1^ (mmHg)	97.8 ± 8.1	102.4 ± 8.6	***p*** **= 0.003**	97.8 ± 10.8	95.7 ± 8	*p* = 0.35
Nightime PP^1^ (mmHg)	65.9 ± 10.2	66.4 ± 10.9	*p* = 0.7	59.2 ± 10.9	57.8 ± 13.6	*p* = 0.46
Nightime BP load SBP^1^ (%)	59.8 ± 21.1	68.5 ± 19.2	***p*** **= 0.04**	51.5 ± 27.5	43.5 ± 26.7	*p* = 0.09
Nightime BP load DBP^2^ (%)	24 (17–38)	37 (20–43)	***p*** **= 0.002**	21 (5–32)	12 (0–29)	*p* = 0.19

BP, blood pressure; SBP, systolic blood pressure; DBP, diastolic blood pressure; PP, pulse pressure; MAP, mean arterial pressure.

^a^Mean ± standard deviation.

^b^Median with interquartile range (IQR).

Significant values are in bold.

The percentages of values above a reference limit (readings > 135/85 mmHg during awake period and 120/70 mmHg during sleeping period) were significantly increased after liraglutide compared to placebo in patients with eGFR < 30 ml/min/1.73 m^2^, whereas no significant change was observed in patients with less impaired kidney function (Table 2).

**Figure 1:**
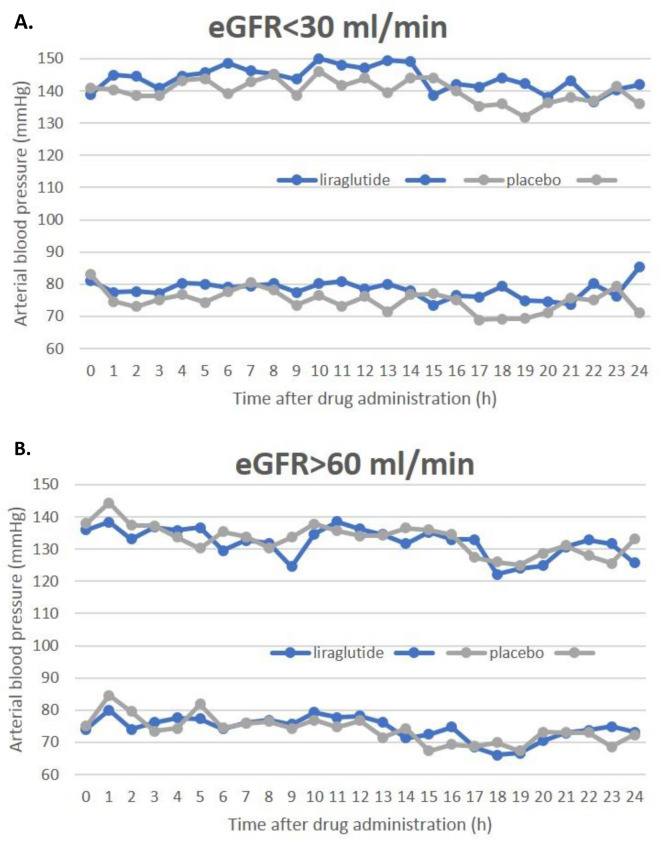
24-h ambulatory systolic blood pressure and diastolic blood pressure after liraglutide and placebo in patients with eGFR < 30 ml/min (**A**) and > 60 ml/min (**B**).

**Figure 2:**
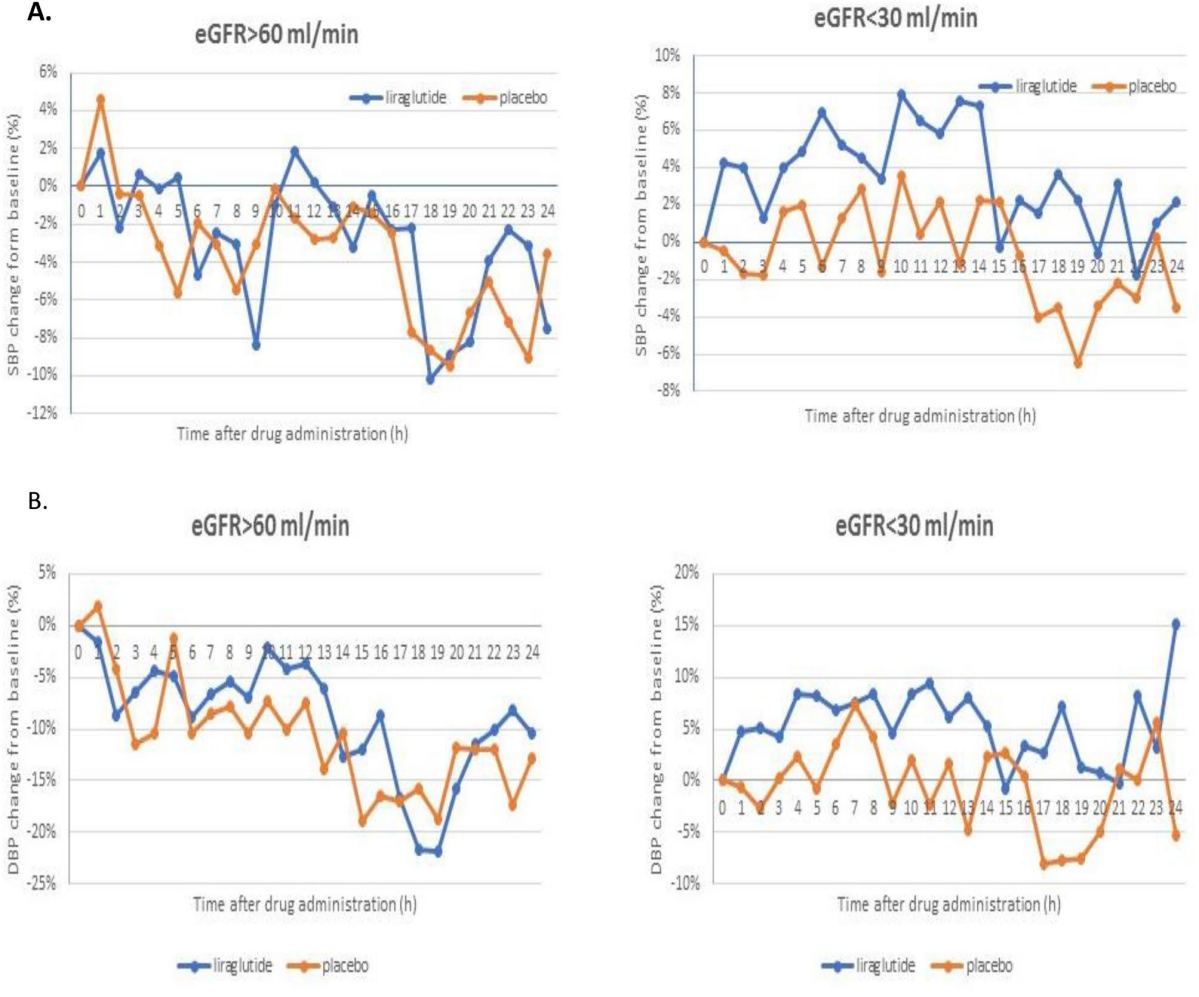
Percentage changes form baseline of 24-h systolic (**A**) and diastolic (**B**) blood pressure after liraglutide and placebo in patients with eGFR < 30 ml/min and > 60 ml/min.

Figure 1 displays graphically 24-h ABPM profiles and Fig. 2 demonstrates relative, percentage changes of blood pressure after liraglutide and placebo from baseline in both study groups. After liraglutide the tendency to higher hourly averaged blood pressure, especially systolic pressure values, were observed in patients with eGFR < 30 ml/min/1.73 m^2^.

The changes of nighttime mean arterial pressure after liraglutide and placebo were comparable, while the morning surge was significantly reduced after liraglutide compared to placebo only in patients with eGFR > 60 ml/min/1.73 m^2^ (Table 3).

**Table 3:** Night blood pressure fall (NBPF), morning surge (MS) and smoothness index (SI) after injection of liraglutide and placebo in the patients with eGFR < 30 ml/min and > 60 ml/min.

eGFR < 30 ml/min/1.73m^b^				eGFR > 60 ml/min/1.73m^b^			
	Placebo	Liraglutide	*p*-value		Placebo	Liraglutide	*p*-value
NBPF^a^ (%)	4.7 ± 8.3	3.6 ± 8	*p* = 0.81	NBPF^a^ (%)	6.4 ± 9.2	7.3 ± 7.8	*p* = 0.63
MS^a^ (mmHg)	9.3 ± 17.1	15.7 ± 9.3	*p* = 0.15	MS^a^ (mmHg)	22.6 ± 14.3	15 ± 14.5	***p*** **= 0.04**
ADI^b^	16.3 (13.9–17.4)	15.1 (11.2–18.2)	*p* = 0.41	ADI^2^	16.8 (14.1–19.2)	16.2 (13–18.5)	*p* = 0.65

NBPF, night blood pressure fall; MS, morning surge; ADI, average deviation index.

^a^Mean ± standard deviation.

^b^Median with interquartile range (IQR).

Significant values are in bold.

The significant increases of 24 h mean heart rate were seen in both groups. In the patients with eGFR > 60 ml/min/1.73 m^2^ mean 24 h heart rate was 73 ± 8 after liraglutide compared with 68 ± 5 beats per minute (bpm) after placebo (*p* = 0.005), whereas in patients with eGFR < 30 ml/min/1.73 m^2^ the respective values were 76 ± 9 and 67 ± 9 bpm (*p* < 0.001 ).

**Figure 3:**
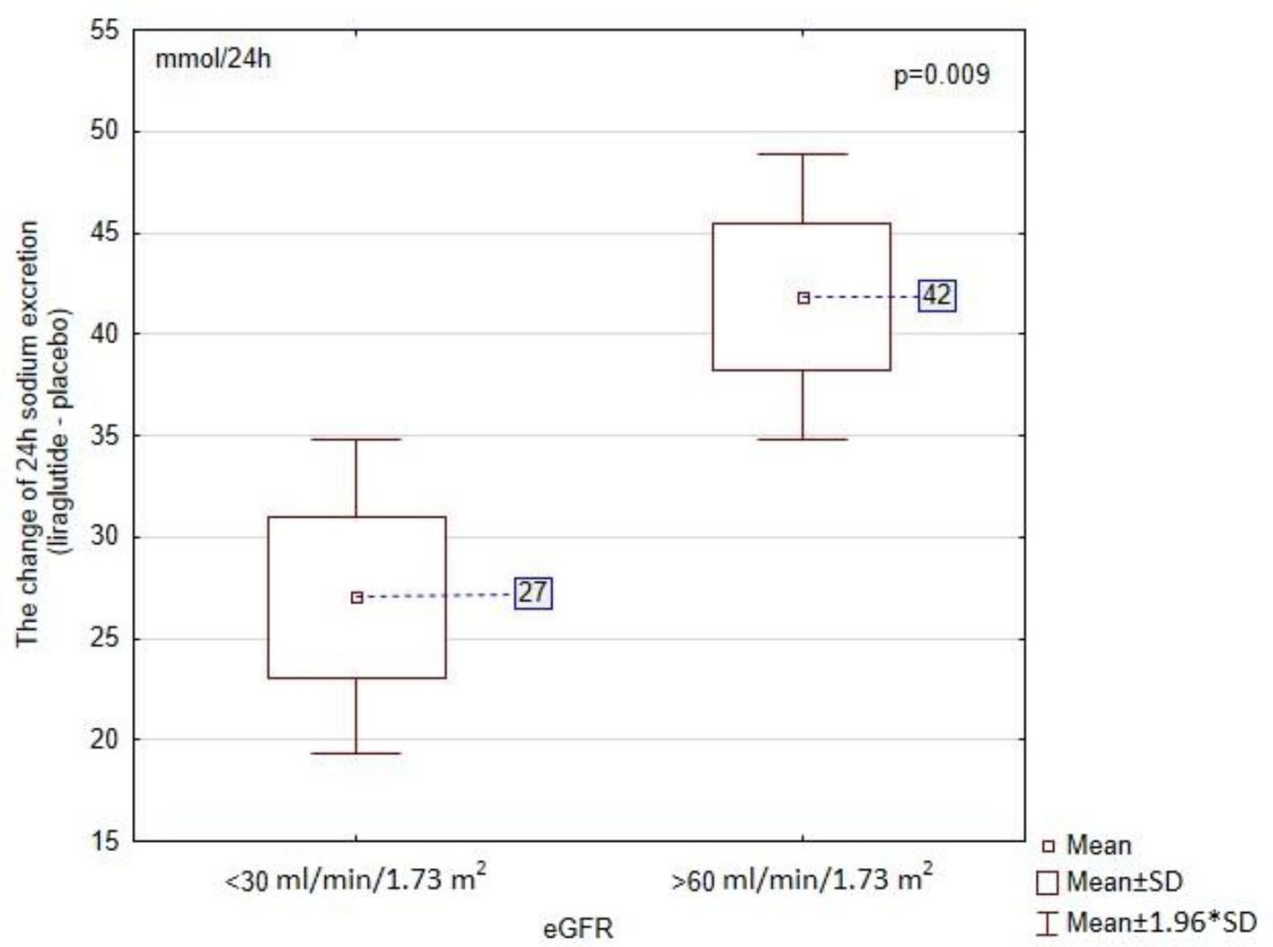
The difference in 24 h sodium excretion (liraglutide-placebo).

After liraglutide mean 24 h urine sodium excretion increased in both groups. The 24 h urine sodium excretion after liraglutide was higher than after placebo in patients with eGFR < 30 ml/min/1.73 m^2^ [242 ± 28 vs. 215 ± 20 mmol/24 h; *p* < 0.001] and in those with eGFR > 60 ml/min/1.73 m^2^ [231 ± 24 vs. 273 ± 29 mmol/24 h; *p* < 0.001]. The difference in natriuretic effect between liraglutide and placebo was shown in Fig. 3. The relative increase of 24 h sodium excretion was significantly larger in patients with eGFR > 60 ml/min/1.73 m^2^ than in patients with eGFR < 30 ml/min (18.2% and 12.6% respectively; *p* = 0.025). The difference of mean 24 h sodium excretion between liraglutide and placebo correlated negatively with the difference of mean 24 h systolic (r = − 0.87; *p* < 0.001) and diastolic pressure (r = − 0.6; *p* = 0.01) in the group with eGFR > 60 ml/min/1.73 m^2^.

The thoracic fluid index significantly decreased at Tmax of liraglutide, i.e. 11 h after the injection of the drug (− 5.2) [95%CI (− 10)–(− 0.4)] compared to placebo (− 0.4) [95%CI (− 3)–(2.2)]; *p* = 0.01 in patients with eGFR < 30 ml/min/1.73 m^2^. In patients with eGFR > 60 ml/min/1.73 m^2^ the changes of thoracic fluid index after liraglutide and placebo were similar (0.2 [95%CI (− 8.9)–(9.2)] and 1.1 [95%CI (− 0.9)–(3.0)], respectively) (*p* = 0.50).

The baseline concentrations of ANP, aldosterone and renin did not significantly differ between study days. The change of serum concentration of atrial natriuretic peptide after the injection of liraglutide was larger than after placebo in both study groups (Table 4). The increase of serum ANP was significantly larger in patients with eGFR < 30 ml/min/1.73 m^2^ than in those with eGFR > 60 ml/min/1.73 m^2^ 23 [95%CI (17.2)–(61.6)] and 9.9 [95%CI (3.8)–(34.2)] respectively, *p* = 0.012).

**Table 4:** Atrial natriuretic (ANP), aldosterone and plasma renin concentration before (baseline) and 11 h (Tmax) after the administration of a single dose of liraglutide and placebo in patients with eGFR < 30 ml/min and > 60 ml/min.

Study group	Intervention	Time of assessment	Hormone		
			ANP (pg/mL)	Renin (pg/mL)	Aldosterone (pg/mL)
eGFR < 30	Placebo	Baseline^a^	74.3 (46.6–91.3)	31.3 (20.2–41.9)	271.6 (267.2–278.9)
		Tmax^a^ (after 11 h)	78.9 (59.8–119.4)	17.2 (13.2–22.1)	266.5 (255.1–279.9)
		*p* value (Baseline vs Tmax)	0.31	**0.0004**	**0.003**
	Liraglutide	Baseline^a^	71.6 (50.4–90.8)	29.6 (20.1–45.9)	278.9 (263.3–284.7)
		Tmax^a^ (after 11 h)	101.2 (87.1–165.8)	16.2 (14.1–20.6)	261.3 (250.4–283.5)
		*p* value (Baseline vs Tmax)	**0.0003**	**0.0004**	**0.003**
	Absolute change after placebo	∆ Placebo^c^	2.6 (− 7.2)–(24.4)	− 8.5 (− 23.6)–(− 2.3)	− 9.4 (− 20.7)–(− 0.2)
	Absolute change after liraglutide	∆ Liraglutide^c^	32.9 (16.4)-(93.5)	− 6.7 (− 19.4)–(− 4)	− 13.8 (− 30.4)–(− 0.4)
		*p* value (liraglutide vs placebo)	**0.0003**	0.54	**0.013**
eGFR > 60	Placebo	Baseline^a^	56.4 (9.7–94.2)	20 (16.8–34.1)	279.5 (271.5–375.5)
		Tmax^a^ (after 11 h)	58.9 (13.7–100.7)	18.4 (16.2–27.4)	275.8 (268.9–286.1)
		p value (Baseline vs. Tmax)	0.4	**0.0005**	**0.017**
	Liraglutide	Baseline^a^	57.1 (9.5–95.7)	20.1 (18.9–30.2)	282.6 (277–374.2)
		Tmax^a^ (after 11 h)	70.5 (16.8–138.1)	19.1 (17.9–29)	279.1 (268.1–285.2)
		*p* value (Baseline vs. Tmax)	**0.003**	**0.003**	**0.02**
	Absolute change after placebo	∆ placebo^c^	− 0.2 (− 5.9)–(2.3)	− 1.3 (− 2.9)–(− 0.7)	− 1.9 (− 16.9)–(− 0.4)
	Absolute change after liraglutide	∆ liraglutide^c^	9.3 (3.4)–(30.8)	− 1.5 (− 2.6)–(− 0.2)	− 2.1 (− 20.2)–(0.9)
		*p*-value (liraglutide vs. placebo)	**0.0003**	0.36	0.5

^a^Median with interquartile range (IQR)).

^b^Mean with 95% confidence interval.

^c^Median with 95% confidence interval ).

Significant values are in bold.

In both study groups a significant decrease of serum aldosterone and plasma renin concentration was observed after placebo as well as after liraglutide injection. The change of aldosterone concentration was significantly larger after liraglutide than after placebo only in patients with eGFR < 30 ml/min/1.73 m2 (*p* = 0.013) (Table 4). The change of plasma aldosterone concentration after the administration of liraglutide corrected for the effect of placebo was significantly larger in patients with eGFR < 30 ml/min/1.73 m^2^ compared to the patients with eGFR > 60 ml/min/1.73 m^2^, (− 7.3) [95%CI (− 11.7)–(− 0.2)] and (− 0.3) [95%CI (− 2.2)–(3.8)] respectively, (*p* = 0.007).

The changes of renin plasma concentration after liraglutide and placebo were similar in both study groups.

## Discussion

Glucagon-like peptide 1 agonists have been found to modestly lower blood pressure in diabetic patients in most of the studies^8,13^. Although the mechanisms leading to a blood pressure decrease during the treatment with GLP-1 agonists have not been fully elucidated it could be the result of an increased sodium and water excretion and/or inhibition of the RAAS activity^14^. In this study we investigated the effect of GLP-1R agonist liraglutide on blood pressure, hydration status, natriuresis and the activity of the renin–angiotensin–aldosterone axis in patients with diabetic kidney disease after a dose of liraglutide compared to placebo. The choice of liraglutide was substantiated by its favorable pharmacokinetic profile in patients with impaired kidney function and therefore by its increasing clinical use in this population^12^. Across studies and populations, liraglutide was found to be slowly absorbed following a subcutaneous injection, with a time to maximum plasma concentration of the drug (Tmax) of approximately 11–12 h^11^. The drug is completely degraded into several non-active metabolites within the body and as was shown in the study with radiolabeled drug, no intact liraglutide is excreted in either urine or feces^11^. What is more, no clear trend for a change in pharmacokinetics was evident across groups with increasing renal dysfunction^12^ that allowed us to administer the same dose of the drug and the same blood sampling time points in patients with different degree of renal impairment who were included in our study.

The main finding of our study is the observation that a degree of renal impairment affects the magnitude of the effect of liraglutide on blood pressure and natriuresis. Contrary to our initial expectations, we did not observe a significant blood pressure lowering effect of liraglutide in patients with mild CKD, ie. with eGFR > 60 ml/min/1.73m^2^. Instead in patients with eGFR < 30 ml/min/1.73m^2^ a single injection of liraglutide led to a significant transient increase of blood pressure.

Seemingly the results of our study contrast with liraglutide in a range of clinical studies. In the metanalyses of many randomized controlled trials with GLP-1 agonists liraglutide and exenatide showed a significant reduction of systolic blood pressure compared to placebo^13,15,16^. The studies included in these metanalyses were performed however in patients with without or with only moderately impaired kidney function. Interestingly, in a few small randomized controlled trials that assessed the safety and efficacy of liraglutide in patients with impaired kidney excretory function, the effect of the drug on blood pressure differed from that observed in patients with normal kidney function or in early CKD. In LIRA-RENAL study, that included 279 patients with moderate renal impairment (eGFR 30–59 mL/min/1.73 m^2^), SBP reduction of 2.45 mmHg was seen but the change was not significantly different between liraglutide and placebo (*p* = 0.25)^17^. In the LIRA-RENAL trial a mean eGFR was however significantly lower than in our patients, the patients had a longer history of diabetes and liraglutide was administered in a 50% higher daily dose of 1.8 mg. Hiramatsu et al. studied a long-term effect of liraglutide in three subgroups of CKD patients with eGFR > 60 ml/min/1.73m^2^, 30–60, and < 30 ml/min/1.73 m^2^^18^. They found that a systolic blood pressure decreased significantly after 24 months of treatment with 0.9 mg/24h compared to baseline in all groups. A small improvement of blood pressure control after liraglutide treatment was also seen in patients with end-stage kidney disease treated by peritoneal dialysis^19^. In contrast to the blood pressure decrease reported by Hiramatsu et al. in patients with CKD including stage 4 with eGFR < 30 ml/min/1.73 m^2^, Indorn et al. did not find any significant change of blood pressure in chronic dialysis patients after 12 weeks of liraglutide treatment, titrated to a maximum dose of 1.8 mg daily^20^. We may speculate that the difference between the results of our study and from most previous studies may primarily be dependent of treatment duration but also may be affected by different methods of blood pressure measurement. In the randomized placebo controlled study of Kumarathurai et al. in patients with type 2 diabetes mellitus and ischemic heart disease, in which ambulatory blood pressure monitoring was used liraglutide did not induce any significant change of mean 24h SBP, whereas the tendency towards blood pressure reduction was observed if office blood pressure was analyzed^21^. In another study there was a small but significant increase of 24-h and nighttime diastolic BP after 3 weeks of liraglutide treatment^22^ similar to the effect of a single dose of the drug in our study. In contrast Liakos et al. found that liraglutide administered for 5 weeks had a favorable effect on 24h systolic but not diastolic blood pressure profile^23^.

The acute effect of liraglutide on blood pressure was investigated in several studies. The studies of Bharucha et al.^24^, Asmar et al.^25^ and Mendis et al.^26^ in healthy volunteers revealed a small but significant increase of blood pressure after a single injection of liraglutide. These results are similar to seen in our study but only in patients with eGFR < 30 ml/min/1.73 m^2^. These results may show that the acute effect of liraglutide may be different that chronic. The difference of the acute and chronic effects of GLP-1R agonist was also demonstrated in a study of von Scholten et al.^27^ that showed 24 h SBP increase during the treatment as high as 10 mmHg after 3 days of treatment (*p* = 0.008). The effect decreased to 7 mmHg on day 7 (*p* = 0.033) and disappeared after 29 days of treatment. At that time 24h SBP decreased and was 7 mmHg lower compared with baseline value. That difference was however not significant (*p* = 0.11).

The cumulative evidence may support a role of GLP-1 in the regulation of fluid and electrolyte balance mediated by the reduction of thirst and increased natriuresis. GLP-1 infusion significantly increased the renal clearance of sodium by about 40% with a simultaneous 20% decrease of sodium reabsorption in the proximal tubule^28^. Gutzwiller et al. observed that an intravenous infusion of native GLP-1 increased sodium excretion from 59 to 96 mmol/180 min (*p* = 0.015) in obese males^29^. A similar significant effect of GLP-1R agonists on sodium excretion was observed in both experimental and clinical studies. The administration of exenatide led to an increase of both absolute and fractional sodium excretion in healthy men in a study of Muskiet et al.^30^ and similar results were reported in the patients with diabetes^31^. Liraglutide significantly increased 24-h and nighttime urine sodium excretion in patients with type 2 diabetes mellitus and arterial hypertension after 3-week treatment^22^ but similar effect was also reported after a single dose of this drug^32^. While our results confirmed the natriuretic effect of liraglutide, the increase of sodium excretion was significantly smaller in the patients with eGFR < 30 ml/min/1.73 m^2^ compared to those with eGFR > 60 ml/min/1.73 m^2^. These finding is not unexpected since GLP-1 may increase natriuresis through the inhibition of many ion exchangers in the tubules which expression decreases with the progression of CKD and interstitial fibrosis and tubular atrophy^33^.

Although the natriuretic effect of liraglutide was small in the patients with eGFR < 30 ml/min/1.73 m^2^ the thoracic fluid index, a marker of hydration status assessed with impedance cardiography significantly decreased. The same effect of liraglutide was revealed in another study in patients with diabetes and hypertension but without renal impairment^27^. The different effect on the hydration status seen in our groups might have been caused by the different baseline hydration status as fluid retention tends to progress with renal impairment^34^.

A significant decrease of plasma aldosterone was seen in our study after liraglutide compared with placebo only in patients with eGFR < 30 ml/min/1.73 m^2^. Skov et al. did not find any significant changes of renin, aldosterone, and urinary angiotensinogen after a single GLP-1 infusion in healthy men^28^ and the same result was reported in patients with diabetes without kidney function impairment^32^. In contrast the decrease of plasma aldosterone was observed by Baretic et al.^35^ and Sedam et al.^36^ in healthy participants. We may speculate that a larger reduction of plasma aldosterone in the patients with advanced CKD seen in our study could be caused by a higher local activity of RAAS due to kidney dysfunction.

After the injection of liraglutide serum atrial natriuretic peptide increased more than after placebo in both study groups. Such effect was not seen in previous studies but most of them were conducted in healthy men. Contrary to our results von Scholten et al. found that after 7 weeks of liraglutide administration serum ANP decreased by 20% (95% CI 12–28%, *p* < 0.001)^27^. A significant increase of ANP level after the administration of a GLP-1 agonist was found mainly in experimental models with hyperreninemia and fluid overload-dependent hypertension^8^. The tendency to fluid retention is highly prevalent in diabetic kidney disease^34^. We suppose that there might be an association between a grade of overhydration and the magnitude of the effect of GLP-1R agonists on atrial natriuretic peptide secretion, that may explain a significantly larger increase of serum ANP concentration in patients with eGFR < 30 ml/min/1.73 m^2^ in our study. However we did not reveal any significant correlation between thoracic fluid index and its change and the change of ANP concentration. The small sample size could be one of possible reasons. We may speculate that since GLP-1 increases natriuresis through an indirect inhibition of the sodium-exchangers in the tubules, which expression decreases in parallel to progressing tubular atrophy in the course of CKD^33^, the GLP-1 receptor agonist administration could result in the smaller increase of natriuresis in the group with eGFR < 30 ml/min despite the increase of ANP, that was observed in our study.

On the other hand a GLP1-ANP axis has been only demonstrated in hypertensive mice, in which GLP-1R activation in the atria leads to ANP secretion and stimulation of natriuresis and vasodilation in the consequence. When comparing data from rodents with humans, results regarding GLP-1’s renal effects are very different.

Sustained liraglutide administration for 3 weeks increases urinary sodium excretion independent of changes in ANP or blood pressure in overweight and obese hypertensive patients with type 2 diabetes^22^. Most likely, the natriuretic effect of GLP-1 in humans is mediated by a tubular mechanism secondary to selective suppression of angiotensin II^28,37^. Asmar et al. recently demonstrated that GLP-1’s natriuretic effect, GLP-1-dependent angiotensin II suppression, and high renal GLP-1 extraction all depend specifically and fully on GLP-1 receptor activation. The study showed that the renal extraction of GLP-1, natriuretic effect of GLP-1 and change of plasma concentrations of ANG II was abolished by the GLP-1 receptor antagonist Exendin 9–39 (Ex 9–39), suggesting that GLP-1-induced natriuresis is dependent on GLP-1 receptor activation^38^. In another study in volume-loaded participants, GLP-1 induced natriuresis together with the ANG II level decrease, independent of renal hemodynamics (renal plasma flow, GFR unchanged) and renin concentrations^37^. The angiotensin II evaluation was also planned in our protocol, but measurements were not finally performed due to technical difficulties in our research laboratory.

Despite the limitations like small sample size, the recruitment limited to two stages of DKD, the assessment of only an acute effect of the drug our study found a short-term hypertensive effect of GLP-1R agonist liraglutide in diabetic kidney disease that seems to be related to impaired renal sodium excretion. The natriuretic effect may not sufficiently counteract the initial increase of blood pressure in patients with severe kidney function impairment. We also postulate that increased natriuretic after liraglutide in severe diabetic kidney disease may result from increased atrial natriuretic peptide secretion and decreased aldosterone secretion. The mechanism of these changes will however require further research.

## Data Availability

The datasets used and/or analyzed during the current study available from the corresponding author on reasonable request.
